# Multiple dimensions underlying the functional organization of the language network

**DOI:** 10.1016/j.neuroimage.2021.118444

**Published:** 2021-11-01

**Authors:** Victoria J. Hodgson, Matthew A. Lambon Ralph, Rebecca L. Jackson

**Affiliations:** MRC Cognition and Brain Sciences Unit, University of Cambridge, UK

**Keywords:** Semantics, Phonology, Meta-analysis, Control, Language, Multiple Demand Network

## Abstract

Understanding the different neural networks that support human language is an ongoing challenge for cognitive neuroscience. Which divisions are capable of distinguishing the functional significance of regions across the language network? A key separation between semantic cognition and phonological processing was highlighted in early meta-analyses, yet these seminal works did not formally test this proposition. Moreover, organization by domain is not the only possibility. Regions may be organized by the type of process performed, as in the separation between representation and control processes proposed within the Controlled Semantic Cognition framework. The importance of these factors was assessed in a series of activation likelihood estimation meta-analyses that investigated which regions of the language network are consistently recruited for semantic and phonological domains, and for representation and control processes. Whilst semantic and phonological processing consistently recruit many overlapping regions, they can be dissociated (by differential involvement of bilateral anterior temporal lobes, precentral gyrus and superior temporal gyri) only when using both formal analysis methods and sufficient data. Both semantic and phonological regions are further dissociable into control and representation regions, highlighting this as an additional, distinct dimension on which the language network is functionally organized. Furthermore, some of these control regions overlap with multiple-demand network regions critical for control beyond the language domain, suggesting the relative level of domain-specificity is also informative. Multiple, distinct dimensions are critical to understand the role of language regions. Here we present a proposal as to the core principles underpinning the functional organization of the language network.

## Graphical abstract

**Figure:**
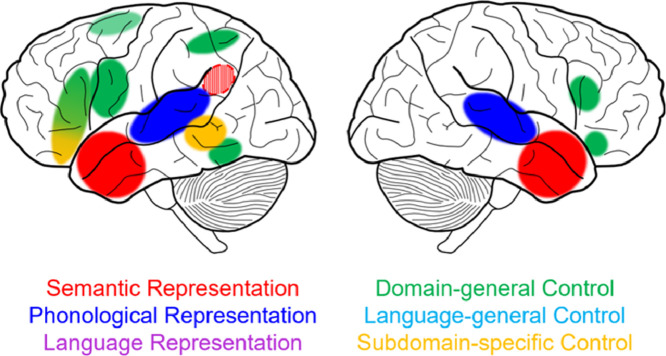

## 1 Introduction

The complexity of language and its neural substrates have long drawn attention within cognitive neuroscience. Though it remains unclear how precisely language is organized in the brain, different facets of language can be dissociated from one another at the behavioral and neuropsychological levels; in particular, a key division between semantics and phonology (Démonet et al., 1992, Geschwind, 1970, Vigneau et al., 2006, Vigneau et al., 2011). Yet questions remain as to whether these processes are supported by different neural networks, and to what degree, and in what manner, these networks interact. Moreover, semantics and phonology may each be supported by multiple interactive networks, underpinning dissociable processes at the cognitive level. Process-based divisions, such as the separation between representation and control processes previously specified within the domain of semantic cognition (Lambon Ralph et al., 2017, Rogers et al., 2004, Jefferies, 2013), may provide additional, and perhaps orthogonal, information regarding the functional role of regions of the language network. To investigate whether these domain- and process-based divisions delineate the functional roles of regions across the language network, we conducted a series of neuroimaging meta-analyses to establish the patterns and locations of consistent activation within and across three domains (semantic cognition, phonological processing and non-verbal working memory) and across varying levels of control demands (identifying regions specialized for representation versus control processes).

The neural correlates of language may differ based on the importance of semantic versus phonological demands. Phonological processing is the perception, analysis and use of language sounds to understand and produce spoken and written language (Poldrack et al., 1999, Wagner and Torgesen, 1987). The production and perception of phonological information converge in the temporoparietal junction, involving posterior superior temporal lobe, inferior Superior Marginal Gyrus (SMG), and left Inferior Frontal Gyrus (IFG) (Buchsbaum et al., 2001, Hickok and Poeppel, 2007, Buchsbaum et al., 2011, Graves et al., 2008, Démonet et al., 1996, Zatorre et al., 1996). Semantic cognition refers to the storage, retrieval and manipulation of multimodal conceptual knowledge. A distributed network of regions are implicated in multimodal semantic cognition, including IFG, posterior temporal cortex, Inferior Parietal Lobe (IPL) and Anterior Temporal Lobe (ATL). The ATL acts as a multimodal hub mediating between numerous modality-specific regions, or “spokes” distributed across sensory and association cortices (Lambon Ralph et al., 2017, Rogers et al., 2004, Jefferies, 2013, Patterson et al., 2007, Patterson and Lambon Ralph, 2016, Binder and Desai, 2011, Binder et al., 2009). Neighboring and potentially overlapping areas of the left posterior temporal lobe, IPL and IFG have been implicated in both the semantic and phonological networks; some of these apparent similarities, however, may be obscuring graded differences in specialization for each language domain, and there is a need for a more direct comparison. For example, neuroimaging provides some evidence of relative functional specialization for semantic cognition in more ventral and caudal, and phonology in more dorsal, rostral left IFG (Poldrack et al., 1999, Fiez, 1997). Transcranial magnetic stimulation induced virtual lesions of dorsal left IFG impair performance on phonological tasks (Aziz-Zadeh et al., 2005, Nixon et al., 2004), while disrupting the ventral left IFG diminishes semantic performance (Devlin et al., 2003, Köhler et al., 2004). Left IFG may therefore constitute two specialized sub-regions for different aspects of language, or a single complex with graded functional specialization (Devlin and Watkins, 2007). Similarly, the IPL is not a homogenous region, with debate as to which sub-regions are implicated in semantic and phonological processing and beyond language (Humphreys et al., 2015, Cabeza et al., 2012, Seghier, 2013, Noonan et al., 2013, Bzdok et al., 2016).

**Fig. 1:**
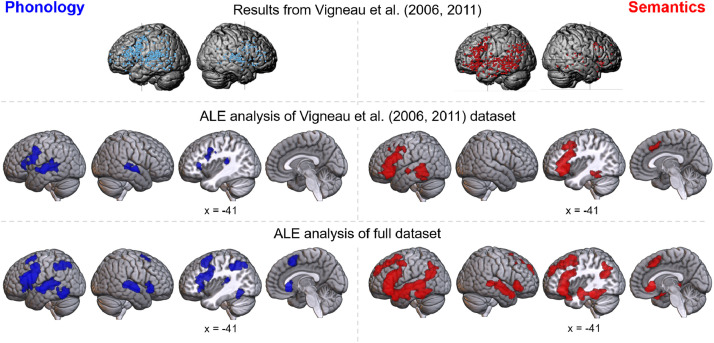
Results for the phonology and semantics domains are shown in blue (left column) and red (right column) respectively. Top row: original results by Vigneau et al., 2006, Vigneau et al., 2011; figures are reproduced with permission from Vigneau et al. (2011). Middle row: formal ALE meta-analysis of the recreated Vigneau et al., 2006, Vigneau et al., 2011 datasets, with 615 foci from 44 experiments for phonology, and 788 foci from 70 experiments for semantics. Bottom row: meta-analysis of full datasets, with 1176 foci from 82 experiments for phonology, and 2819 foci from 209 experiments for semantics.

The neural correlates of phonology and semantics were the subject of a seminal review of organization across the language network by Vigneau et al., 2006, Vigneau et al., 2011. The authors identified neuroimaging studies targeting semantics, phonology and sentence processing and mapped the peak group activations (see Figure 1). Both domains resulted in highly distributed networks of peaks principally centered on the left hemisphere. The distribution of peaks clustered upon bilateral precentral, superior temporal and inferior frontal gyri, and left posterior temporal lobe and supramarginal gyrus for phonology, whilst implicating bilateral IFG, posterior inferior and superior temporal gyri and left Angular Gyrus (AG), planum temporale and ATL in semantics. Based on visual examination, Vigneau et al., 2006, Vigneau et al., 2011 concluded the distribution of peaks reflected distinct networks for phonology and semantic cognition. As this was published prior to the widespread use of formal neuroimaging meta-analyses (Müller et al., 2018), it was not possible to determine which regions were significantly consistently involved, or to directly contrast the areas implicated in semantic and phonological processing. Despite these unavoidable limitations, the paper continues to be highly cited as evidence of a strong dissociation between the neural correlates of semantics and phonology. Accordingly, in this investigation we apply formal Activation Likelihood Estimation (ALE) analyses and update the neuroimaging database, to determine the regions involved in phonological and semantic cognition and directly contrast the likelihood of activation in these two subdomains of language.

In the years since Vigneau et al., 2006, Vigneau et al., 2011 reports, an additional process-based distinction has gained increasing recognition, both within the language domain and in cognition more broadly. Alongside the representation of information relevant for each domain, executive control processes are critical for the context-appropriate access and use of this information (Lambon Ralph et al., 2017, Badre et al., 2005, Duncan, 2010, Jefferies and Lambon Ralph, 2006). The crucial nature of the division between control and representation processes has been highlighted for semantic cognition, where the Controlled Semantic Cognition (CSC) framework proposes relative functional specialization of regions for representation versus control, resulting in distinct neural architectures (Lambon Ralph et al., 2017, Jackson et al., 2019, Rogers et al., 2015). Semantic control refers to the contextual selection and manipulation of semantic information necessary for task-appropriate behavior, particularly when dominant features must be supressed, or subordinate meanings must be accessed (Jefferies, 2013, Badre et al., 2005, Davey et al., 2016). A subset of the regions involved in general semantic processing are specifically implicated in semantic control: the IFG, left posterior lateral temporal lobe (specifically, posterior middle and inferior temporal gyri) and bilateral dorsomedial prefrontal cortex (dmPFC) (Jefferies, 2013, Noonan et al., 2013, Jefferies and Lambon Ralph, 2006, Jackson, 2021, Noonan et al., 2009, Whitney et al., 2011, Whitney et al., 2011).

Like semantic cognition, phonology is supported by control processes, though these have not been emphasized to the same extent, nor have their neural correlates been clearly isolated to date. However, some functional neuroimaging work has found activation in left IFG, precentral gyrus, SMG and dmPFC for controlled, effortful phonological tasks (e.g., tasks in which participants must regularize the pronunciation of irregular words) (Noonan et al., 2013, Gold et al., 2005, Gold and Buckner, 2002). To determine whether control versus representation is a critical organizational principle of the language network, this investigation used meta-analyses to isolate regions critical for phonological and semantic control. Control regions could be particular to a subdomain of language, specific to the broader domain of language, or domain general. Therefore, considering an additional ‘domain-specificity’ factor could help further elucidate the organization of the language network. This may be tested by assessing whether the same regions are implicated in semantic control, phonological control and additional analyses targeting domain-general control. The Multiple Demand Network (MDN) is a set of brain regions activated across a broad range of executive tasks, indicating support for a wide range of tasks across domains (Duncan, 2010), and includes the posterior inferior frontal sulcus, intraparietal sulcus, anterior insula, pre-supplementary area and anterior cingulate cortex (Duncan, 2010, Assem et al., 2019, Camilleri et al., 2018). This may overlap regions implicated in phonology (such as inferior parietal cortex (Humphreys et al., 2015, Pollack and Ashby, 2018)) or semantics. Although largely distinct, the semantic control areas identified in Jackson’s (2021) meta-analysis of semantic control may overlap Assem et al.,’s (2020) extended MDN in a portion of the IFG and left posterior inferior temporal cortex.

There were three steps to the present study. Firstly, formal ALE analyses compared the regions implicated in the semantic and phonological subdomains, testing the supposition that these subdomains rely on distinct neural correlates. To determine the relative impact of changing method of analysis versus dataset, this was first performed using Vigneau et al’s (2006, 2011) dataset, and then with updated semantic and phonological datasets. Next, ALE analyses of phonological control and semantic control were compared to the full results for phonological and semantic cognition, to determine the importance of control versus representation processes in these language subdomains. Finally, the specificity of the identified control regions, was considered through comparison to two activation maps of domain-general control: an ALE analysis of the *n*-back working memory task (a formal analysis allowing careful control including the elimination of verbal stimuli); and the multiple demand network map from Fedorenko et al. (2013) (used as a standard measure of the MDN, reflecting a larger breadth of executive tasks). Control regions could be involved in one particular language subdomain, language in general or all cognition.

## 2 Methods

Activation Likelihood Estimation (ALE) meta-analyses were conducted for the semantic and phonology domains, independently for Vigneau et al.’s data and the full datasets, which were compared to one another using formal contrast analyses. Subsequently, formal ALE analyses were conducted for semantic control, phonological control, and the *n*-back working memory task.

### 2.1 Inclusion and Exclusion Criteria

All meta-analyses included only peer-reviewed English language articles, describing task-based fMRI and PET studies that reported peak coordinates of a univariate contrast in standard space (Talairach or MNI) and focused on a young healthy adult sample (below 40 years old). Contrasts were excluded if they focused on patients, clinical trials or individual differences (e.g., age, gender, native language). Finally, any contrasts that overlapped with the other domains – e.g., phonological working memory tasks – were excluded, to allow for meaningful comparisons to be made between domains. Within each paper, wherever multiple task contrasts were reported for the same participant sample, all the peak activation coordinates were analyzed as a single contrast, following the recommendation from Müller et al. (2018).

For each domain, studies were sourced from one or more existing published meta-analyses, providing appropriate inclusion and exclusion criteria in keeping with the accepted definition of that particular domain. As the phonology meta-analyses alone did not bring the literature included up-to-date, the same criteria were applied to a literature search. This gained more data and allowed fair comparison with the semantics domain. In addition, each contrast had to meet the general inclusion criteria provided above. For semantics and phonology, formal analyses were conducted employing the data from Vigneau et al., 2006, Vigneau et al., 2011 including both the left and right hemisphere peaks. For these datasets, the original inclusion and exclusion criteria were kept; in total, this included 44 experiments for phonology, and 70 experiments for semantics.

The studies for the semantics domain (Supplementary Table 2) were taken from a meta-analysis by Jackson (2021), which included 272 verbal and nonverbal contrasts that specifically compared a semantic condition with a non-semantic (or less semantic) condition. This included contrasts that compared semantic > less semantic tasks, semantic > non-semantic tasks and meaningful/known > non-meaningful/unknown stimuli. Comparison of high > low familiarity or imageability were excluded, as were studies that used rest or fixation as a baseline, as it has been shown that subtraction of low-level baselines is likely to remove semantic activations due to the high level of semantic processing during rest (Visser et al., 2009). Contrasts containing non-verbal semantic stimuli were also removed, to restrict the focus of the present investigation to the language network, resulting in a dataset of 2819 foci from 209 experiments.

To determine which semantic regions are involved in control, an additional semantic control assessment was included from Jackson (2021)). The original analysis included 96 experiments contrasting more controlled/harder semantics over less controlled/easier semantics, and included tasks that manipulated variables such as homonym ambiguity, interference from competitors and strength of semantic association. Again, contrasts including non-verbal stimuli were removed. The final dataset for semantic control comprised 875 foci from 86 experiments (Supplementary Table 4).

For the phonological domain, studies were sourced from two existing meta-analyses, Vigneau et al., 2006, Vigneau et al., 2011 and Humphreys and Lambon Ralph (Humphreys et al., 2015), which reported peaks for phonology > non-phonological or less phonological tasks, and a Web of Science (https://clarivate.com/products/web-of-science/) search to extend the timeframe of inclusion. Vigneau et al., 2006, Vigneau et al., 2011 included 44 studies (86 contrasts) across a wide range of phonological tasks, including repetition, listening, reading or attending to syllables, letters, pseudo-words and words, judging rhyme, and phonological working memory. 27 papers were taken from Humphreys and Lambon Ralph (Humphreys et al., 2015), primarily contrasts that compared phonological tasks > semantic or orthographical tasks and reported peaks in the parietal lobe. Six contrasts which explicitly investigated working memory were excluded, in order to eliminate conceptual overlap with the *n*-back working memory ALE analysis. Coverage from these two meta-analyses ended in 2009, therefore the Web of Science search was conducted between 2010-2021. Search terms were ‘phonology’ or ‘phonological’ in conjunction with ‘MRI’ or ‘PET’, resulting in 316 results that were assessed for their fit to the inclusion criteria. This yielded an additional 306 foci from 18 experiments that met the inclusion criteria. The final dataset included 82 experiments with a total of 1176 foci (Supplementary Table 3). It was necessary to assess phonological control differently to semantic control, due to the dearth of studies directly focusing on controlled phonological processing. In lieu of a dedicated ALE analysis, the dataset for the phonological domain was divided into hard versus easy tasks, for subsequent formal ALE contrast. Tasks that were passive or simply required a straightforward stimulus-response mapping, e.g., repetition or listening, were classed as easier, while those that involved decision-making, such as judgement of rhyme or syllable number, were classified as harder. In total, 26 experiments with 376 foci in total were included for easy phonology, and 57 experiments with 800 foci in total for hard phonology.

The studies for the *n*-back domain were sourced from Wang et al. (2019). Wang et al. (2019) included 96 published fMRI studies of healthy adults completing verbal, nonverbal, spatial and nonspatial variants of the n-back working memory task, with an *n* between 0 and 3. For the purposes of the present study, only those contrasts that reported a higher > lower *n*-value and met the general inclusion criteria were included. A total of 11 contrasts that contained meaningful (e.g., words or faces) or phonological stimuli were also excluded due to the potential overlap with semantic and phonological processing. The final dataset for this domain included 66 experiments with a total of 1216 foci (Supplementary Table 5).

### 2.2 Activation Likelihood Estimation (ALE) Analyses

Meta-analyses were performed using activation likelihood estimation (ALE) performed in GingerALE version 3.0.2 (https://brainmap.org/ale/; Eickhoff et al., 2009, Eickhoff et al., 2012, Eickhoff et al., 2017, Turkeltaub et al., 2002). All analyses were performed in MNI152 space; before running the analyses, all peaks given in Talairach space were converted to MNI space within GingerALE. ALE is a meta-analytic technique that maps the statistically significant convergence of activation probabilities between experiments considered to reflect similar processes. This is achieved by modeling all foci for each experiment as Gaussian probability distributions, with a full width at half maximum (FWHM) for each Gaussian determined by the sample size of the study (i.e., larger samples result in less uncertainty of the peak's location) to produce a modeled activation map for each experiment included in the analysis. These maps are then merged and ALE scores computed on a voxel-by-voxel basis, with each ALE score representing the probability of an activation being present at that given voxel. For all meta-analyses, ALE scores were thresholded with a voxel-wise p-value of .001. Cluster-level family-wise error correction at a p-value of .001, with 10000 permutations, was then applied to determine minimum significant cluster size and remove non-significant clusters. Formal ALE meta-analyses were conducted for the semantics, phonology and *n*-back domains, for semantic control and for the Vigneau semantics and phonology datasets. The activation maps for the semantics and phonology domains and for hard and easy phonology were also directly contrasted using GingerALE, creating pairwise thresholded conjunction and subtraction ALE images using a *p*-value of .001 with 10000 permutations and a minimum cluster volume of 20 mm^3^.

## 3 Results

Meta-analyses were employed to ask several questions about the neural substrates of language; 1) which brain regions are consistently activated across studies employing semantic and phonology tasks, 2) how distinct are the networks for semantic and phonological processing, 3) does control versus representation provide an additional informative way to separate the functions of regions in the language network and 4) how domain-specific or domain-general are the control areas implicated in semantic and phonological tasks? In considering the division between the neural correlates of semantic cognition and phonology, we first consider whether a formal ALE meta-analysis would reveal this division in the datasets employed by Vigneau et al., 2006, Vigneau et al., 2011, before employing updated datasets to assess whether a division can be identified with the use of both modern methods and up-to-date data.

### 3.1 Separating the Language Network by Phonological and Semantic Subdomain

The formal meta-analysis of the Vigneau et al. (2006, 2011) phonology dataset revealed three clusters (Table 1, Fig. 1). One cluster encompasses mid and posterior Superior Temporal Gyrus (STG), extending into the Sylvian fissure and posterior Middle Temporal Gyrus (MTG). The right hemisphere analogue of this cluster was less extensive, comprising most of the middle to posterior STG with limited expansion into the superior temporal sulcus (STS). An additional left hemisphere cluster lay within the left precentral and inferior frontal gyri (including pars opercularis and triangularis). The formal ALE analysis of Vigneau et al.’s semantic dataset was almost entirely left-lateralized (Fig. 1, peaks provided in Table 2). A large cluster covered left IFG (pars opercularis, triangularis and orbitalis), extending partially into middle frontal and precentral gyri. Another was centered in the posterior MTG, extending dorsally into the STG and ventrally/medially into the inferior temporal gyrus (ITG) and fusiform. The final cluster was located toward the midline, in the left dmPFC, pre-supplementary motor area and paracingulate gyrus.

**Table 1:** Phonology activation likelihood using Vigneau dataset

Cluster	Region of Activation	Maximum ALE Value	Z Score	Peak MNI Coordinate		
				x	y	z
1*	Left superior temporal gyrus/middle temporal gyrus	0.039	6.976	-62	-14	-4
		0.038	6.889	-60	-22	2
		0.025	5.038	-58	-4	-4
		0.024	4.980	-42	-32	16
		0.024	4.926	-64	-34	4
2	Left precentral gyrus/inferior frontal gyrus	0.038	6.902	-50	6	22
		0.034	6.352	-48	4	44
		0.026	5.231	-44	30	10
		0.022	4.574	-50	16	12
		0.016	3.633	-36	24	-2
3	Right superior temporal gyrus/middle temporal gyrus	0.035	6.492	62	-10	-4
		0.029	5.702	62	-30	2
		0.017	3.877	52	-26	8
		0.016	3.713	52	-18	6

⁎ First five peaks only are shown for this cluster

**Table 2:** Semantic activation likelihood using Vigneau dataset

Cluster	Region of Activation	Maximum ALE Value	Z Score	Peak MNI Coordinate		
				x	y	z
1*	Left inferior frontal gyrus/middle frontal gyrus	0.051	7.767	-46	18	24
		0.049	7.520	-44	24	18
		0.041	6.622	-44	22	-2
		0.034	5.747	-38	36	-6
		0.034	5.709	-44	22	-10
2*	Left middle temporal gyrus/superior temporal gyrus/fusiform gyrus	0.043	6.844	-60	-44	-2
		0.034	5.720	-46	-36	-14
		0.032	5.523	-54	-44	8
		0.030	5.288	-54	-52	-10
		0.030	5.285	-58	-14	-4
3	Right dorsomedial prefrontal cortex	0.044	6.953	-2	16	44
		0.019	3.369	12	26	32
		0.018	3.498	-14	30	46

⁎ First five peaks only are shown for this cluster

**Fig. 2:**
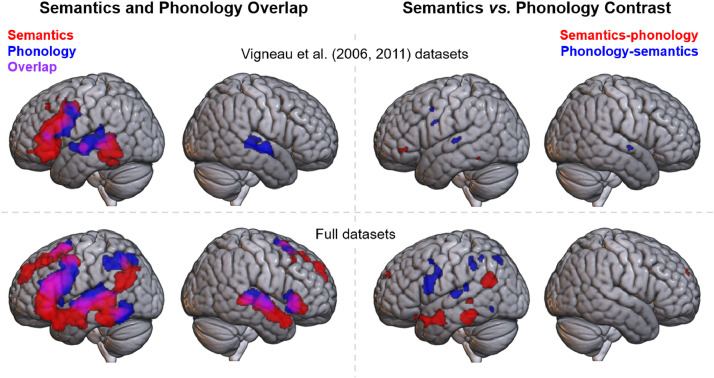
Top row: a comparison of the formal ALE analyses conducted for Vigneau et al.’s semantics (red) and phonology (blue) datasets. Bottom row: a comparison of the formal ALE analyses conducted on the full datasets. Left column: the semantic and phonological activation maps are shown overlaid. Overlap may be seen in violet. Right column: formal ALE contrasts (phonology > semantics in blue, semantics > phonology in red).

Directly contrasting Vigneau et al.’s semantic and phonology datasets (Fig.2, see also Table 5) revealed two clusters for the semantics > phonology analysis, located in the left fusiform gyrus and left pars triangularis, and four clusters for phonology > semantics, located in each middle STG, left precentral gyrus, and left inferior precentral sulcus. All of these clusters were small, with none being larger than 400 mm^3^. Whilst these clusters may reflect true differences between semantics and phonology, these small differences do not provide strong evidence of distinct networks for phonological and semantic cognition.

In comparison, the analyses of the larger, updated phonology and semantic databases generated more extensive networks. For phonology (Fig. 1, peaks given in Table 3), there are seven clusters in total. Three of these are analogous, although more extensive, to the clusters identified with the Vigneau dataset: a large cluster encompassing left IFG (pars opercularis and some of pars triangularis) and a large swathe of precentral gyrus, and one cluster encompassing middle and posterior STG and extending ventrally into the STS in each hemisphere (albeit with a greater extent on the left). In addition, a cluster is located in the left SMG and superior parietal lobule, recruiting a small amount of the AG. Further clusters were located in the left posterior inferior temporal gyrus, right inferior frontal gyrus and near the midline in the left dmPFC. The full semantic activation map, shown in Fig. 1 (peaks given in Table 4), is considerably more extensive than the Vigneau result. A single, large cluster extends across the length of left temporal lobe, a large portion of lateral frontal cortex, insula and ventral aspects of the parietal lobe. The frontal contribution includes the inferior frontal, precentral and middle frontal gyri. In the temporal lobe, the cluster spans from the temporal pole to the planum temporale, recruiting STG, MTG and posterior inferior temporal, fusiform and parahippocampal gyri. This single cluster encompasses and extends beyond the two left lateral temporal clusters revealed in the Vigneau analysis. The second semantic cluster is located in the dmPFC, analogous to, yet more extensive than, the dmPFC cluster for Vigneau et al.’s data. Finally, the full semantic ALE analysis shows right hemisphere activation not revealed using the Vigneau dataset: one cluster encompassing the STG across the length of the temporal lobe, with some involvement of MTG, and a second in the IFG. It is possible that some of this activity is specifically related to sentence-level, syntactic or combinatorial processing and not semantic cognition *per se*; however, when all contrasts featuring sentences or phrases are removed, the resulting ALE map still implicates the same regions with the exception of the right ATL (Supplementary Figure 1). This region may be involved in syntactic processing or may simply fail to reach sufficient power with fewer studies despite a semantic role (in keeping with Rice et al., 2015).

**Table 3:** Phonology activation likelihood

Cluster	Region of Activation	Maximum ALE Value	Z Score	Peak MNI Coordinate		
				x	y	z
1*	Left precentral gyrus/inferior frontal gyrus	0.082	9.806	-50	10	20
		0.072	8.984	-52	16	16
		0.066	8.367	-50	20	22
		0.051	6.990	-50	4	46
		0.046	6.460	-48	30	14
2*	Left posterior superior temporal gyrus	0.055	7.304	-62	-24	4
		0.049	6.742	-60	-16	-2
		0.045	6.303	-62	-32	6
		0.029	4.561	-40	-34	16
		0.028	4.396	-56	-48	8
3	Right posterior superior temporal gyrus	0.044	6.218	62	-10	-2
		0.044	6.206	60	-30	2
		0.024	3.794	46	-24	10
4	Left inferior parietal lobule	0.042	6.062	-30	-56	52
		0.036	5.320	-40	-44	42
		0.031	4.811	-22	-70	50
		0.030	4.618	-46	-42	46
5	Left posterior inferior temporal gyrus	0.038	5.586	-50	-54	-18
		0.037	5.512	-46	-64	-10
		0.022	3.552	-50	-46	-4
6	Dorsomedial prefrontal cortex	0.045	6.284	0	18	52
		0.032	4.917	-2	6	62
7	Right inferior frontal gyrus/insula	0.055	7.325	36	24	-6
		0.023	3.717	50	16	4

⁎ First five peaks only are shown for this cluster

**Table 4:** Semantic activation likelihood

Cluster	Region of Activation	Maximum ALE Value	Z Score	Peak MNI Coordinate		
				x	y	z
1*	Left temporal lobe	0.151	12.526	-56	-38	2
		0.135	11.471	-56	-6	-14
		0.129	11.102	-50	24	14
		0.128	11.022	-50	30	6
		0.122	10.594	-48	20	22
2*	Right superior/middle temporal gyrus	0.081	7.673	54	2	-18
		0.070	6.792	52	-34	0
		0.064	6.300	62	-8	-4
		0.063	6.208	48	14	-26
		0.046	4.741	52	-18	-8
3*	Left dorsomedial prefrontal cortex	0.090	8.382	-4	18	50
		0.075	7.175	-8	52	36
		0.067	6.531	-4	8	58
		0.058	5.755	6	20	44
		0.048	4.906	-2	32	40
4	Right inferior frontal gyrus/insula	0.077	7.319	36	26	-2
		0.047	4.778	36	34	-10

⁎ First five peaks only are shown for this cluster

**Table 5:** Formal contrast phonology vs. semantics Vigneau dataset

Cluster	Region of Activation	Z Score	Peak MNI Coordinate		
			x	y	z
*Phonology > semantics*					
1	Left STG	3.239	-63	-24	7
		3.432	-60	-24	6
2	Right STG	3.353	63	-7	-3
3	Left precentral gyrus	3.291	-60	2	27
4	Left precentral sulcus/gyrus	3.719	-50	4	42

*Semantics > phonology*					
1	Left IFG (pars triangularis)	3.432	-45	38	-5
		3.239	-41	43	-6
2	Left fusiform/parahippocampal gyri	3.432	-38	-48	-16

Contrasting the full phonology and semantic activation likelihood maps revealed significant differences between the networks supporting the two domains (see Figure 2 & Table 6). Greater involvement in phonology was found for a large cluster within the frontal lobe, principally in the precentral gyrus, with some involvement of pars opercularis. A number of smaller clusters were located in AG and SMG, together comprising the majority of the parietal cluster implicated in phonology. Finally, differences were identified in the left STG and the left Sylvian fissure with a small cluster in left posterior ITG. Thus, the superior left posterior temporal lobe, the left precentral gyrus, and left superior parietal lobule are consistently recruited for phonology more than semantic cognition. The semantics > phonology map is comprised of six clusters, identifying regions of left parahippocampal and fusiform gyri, left ATL and left ventral AG that were not implicated in phonology. Smaller clusters were located in the left posterior MTG, left IFG (pars orbitalis) and left superior frontal gyrus. Thus, the left ATL, fusiform gyrus, and ventral AG are consistently recruited for semantic cognition to a greater extent than phonological processing. There appear to be some differences in the posterior temporal regions activated by semantics and phonology, with semantics relying on more anterior and dorsal aspects whilst phonology relies on more posterior ITG. Notably, additional activations in the right ATL and the most ventral portions of the left IFG were present for the semantic but not phonological subdomain, but these apparent differences did not reach statistical significance.

**Table 6:** Formal contrast phonology vs. semantics

Cluster	Region of Activation	Z Score	Peak MNI Coordinate		
			x	y	z
*Phonology > semantics*					
1	Left precentral gyrus	3.891	-54	8	21
		3.719	-46	-4	30
		3.540	-58	-1	30
		3.432	-56	4	10
2	Left posterior superior temporal gyrus	3.891	-58	-23	8
3	Left supramarginal gyrus	3.891	-42	-41	41
4	Left precuneus	3.891	-19	-71	50
		3.719	-23	-73	46
		3.540	-18	-64	52
5	Left posterior superior temporal gyrus	3.891	-41	-35	14
		3.719	-45	-33	13
		3.432	-42	-34	20
6	Left supramarginal gyrus	3.540	-28	-52	51
		3.432	-36	-52	48
		3.353	-32	-50	48
7	Left precentral gyrus	3.891	-52	7	47
8	Left fusiform gyrus	3.432	-52	-65	-11
*Semantics > phonology*					
1	Left parahippocampal cortex	3.891	-31	-35	-18
		3.540	-24	-8	-18
2	Left anterior middle/superior temporal gyri	3.891	-53	1	-22
3	Left angular gyrus	3.891	-45	-61	24
		3.719	-44	-54	18
4	Left posterior middle temporal gyrus	3.891	-53	-39	0
5	Left superior frontal gyrus	3.891	-11	58	31
6	Left inferior frontal gyrus (pars orbitalis)	3.353	-34	24	-18

The neural correlates of semantics and phonology are dissociable despite considerable overlap between the networks recruited. However, substantial separation was only possible with both formal ALE meta-analyses and the additional data present in the full dataset; application of formal analyses alone was not sufficient. This may be due to the increased power of a larger dataset, or various methodological improvements in more recent studies, such as increased sample sizes, multi-banding (Barth et al., 2016, Feinberg and Setsompop, 2013) or the development of fMRI techniques to reduce signal loss in regions such as the anterior temporal lobe (Visser et al., 2010, Halai et al., 2014, Poser et al., 2006).

### 3.2 Separating the Language Network by Representation vs. Control Processes

**Fig. 3:**
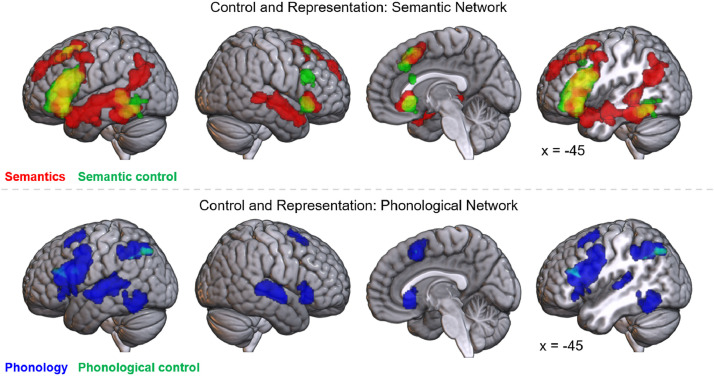
Top row: activation map for semantics domain (red) overlaid with semantic control (green, overlap in yellow). Bottom row: activation map for full phonological domain (blue) overlaid with phonological control, represented by hard > easy phonology formal ALE contrast (green, overlap in cyan).

To determine whether control versus representation is an informative principle of organization of the language network, ALE analyses of semantic and phonological control were conducted. The semantic control analysis identified five clusters (Figure 3 & Table 7). The largest and most significant of these encompasses the entirety of the left IFG, and extends somewhat into the precentral gyrus and orbitofrontal cortex. The second cluster is located in left posterior temporal cortex, namely the posterior MTG and ITG. A third cluster in bilateral dmPFC overlaps with the dmPFC cluster for general semantics, though does not extend as rostrally. In the right hemisphere, two smaller clusters are located in the frontal lobe, one straddling the inferior frontal sulcus, and one more ventrally in IFG (pars orbitalis) and the insula. Control versus representation demands split the semantic network; the IFG, left dmPFC and left posterior temporal cortex form the semantic control network, whereas the remaining regions (bilateral temporal lobe and inferior parietal cortex) likely reflect semantic representation processes.

**Table 7:** Semantic control activation likelihood

Cluster	Region of Activation	Maximum ALE Value	Z Score	Peak MNI Coordinate		
				x	y	z
1*	Left inferior frontal gyrus/precentral gyrus	0.083	9.963	-50	22	20
		0.061	8.054	-48	30	12
		0.060	7.898	-46	24	-2
		0.055	7.489	-50	30	0
		0.037	5.559	-34	26	-6
2	Left dorsomedial prefrontal cortex	0.049	6.877	-2	20	52
		0.033	5.119	2	28	36
		0.029	4.713	-4	30	44
		0.025	4.096	-4	8	58
3*	Left posterior middle/inferior temporal gyri	0.037	5.577	-46	-48	-16
		0.036	5.505	-46	-56	-12
		0.035	5.353	-54	-40	2
		0.034	5.286	-56	-46	-4
		0.021	3.573	-50	-68	-2
4	Right inferior frontal gyrus	0.046	6.583	32	24	-6
		0.019	3.361	30	18	-18
5	Right inferior frontal sulcus	0.042	6.161	52	24	26
		0.028	4.590	40	20	22

⁎ First five peaks only are shown for this cluster

Phonological control, represented by the formal contrast of hard > easy phonology, is displayed in Fig. 3 (also see Table 8). This implicated the inferior parietal lobule (with a cluster spanning supramarginal and dorsal angular gyri and extending medially), the IFG (pars opercularis) and middle frontal gyrus. This does not identify all the regions implicated in both semantic control and phonology, which may be hypothesized to be control regions. However, at a less stringent threshold (voxel-level *p*-value of .01), three additional clusters are revealed in the right fusiform gyrus extending into the cerebellum, the left fusiform gyrus and the right IFG (pars triangularis), and the left frontal cluster extends into the precentral gyrus (see Supplementary Figure 2), indicating that these regions may also reflect control demands in the phonology domain. Like the semantic network, the regions implicated in phonology may be divided on the basis of performing control versus representation processes. Specifically, the bilateral STG may underpin phonological representation, whilst IFG, inferior parietal cortex, precentral gyrus and posterior ITG may all contribute to phonological control.

**Table 8:** Phonological control (hard > easy phonology contrast)

Cluster	Region of Activation	Z Score	Peak MNI Coordinate		
			x	y	z
*Voxel level threshold p* = *.001*					
1	Left precuneus	3.891	-22	-70	46
		3.540	-29	-70	46
2	Left inferior frontal sulcus	3.891	-44	27	20
		3.291	-41	31	24
3	Left inferior frontal gyrus (opercularis)	3.891	-59	12	25
4	Left inferior parietal lobule	3.432	-26	-52	38
		3.239	-32	-56	38
5	Right dorsomedial prefrontal cortex	3.239	8	16	51
6	Left inferior parietal lobule	3.239	-28	-58	40
*Voxel level threshold p* = *.01*					
1	Left inferior frontal/precentral gyrus	2.948	-47	23	20
		3.432	-42	34	22
		3.090	-52	16	20
2	Left precuneus	3.719	-26	-68	44
		3.540	-25	-70	47
		3.432	-24	-76	47
		3.156	-32	-52	36
		3.036	-29	-54	38
3*	Right cerebellum/fusiform gyrus	3.121	36	-58	-26
		0	37	-60	-26
		2.712	32	-76	-16
		2.678	32	-79	-11
		2.628	32	-80	-16
4	Dorsomedial prefrontal cortex	3.540	7	16	52
5	Right inferior frontal gyrus (triangularis)	3.121	42	30	18
		2.989	44	34	14
		2.878	42	26	16
6	Left inferior temporal gyrus	2.759	-40	-64	-8
		2.727	-44	-64	-10
7	Left middle frontal gyrus	2.782	-50	14	38

⁎ First five peaks only are shown for this cluster

### 3.3 Separating the Language Network by Level of Domain-Specificity

Do these control regions respond selectively to specific subdomains of language, subserve language in general, or underpin all cognitive domains? Ventral IFG and posterior MTG were implicated in semantic control only, whilst dorsolateral prefrontal cortex (dlPFC), dorsal IFG (pars opercularis and triangularis), precentral gyrus, dmPFC and posterior ITG were implicated in both semantic and phonological control. These regions may reflect language-general or domain-general control regions. To help to distinguish these two possibilities, we examined the overlap with two measures of domain-general control: an ALE analysis of the working memory *n*-back task and a mask of the multiple demand network (from Fedorenko et al. (2013)). A formal analysis of nonverbal working memory allows rigorous inclusion criterion without any effects of semantic or phonological stimuli, whereas an *a priori* MDN map may provide a more complete picture of domain-general control encompassing different executive functions.

**Fig. 4:**
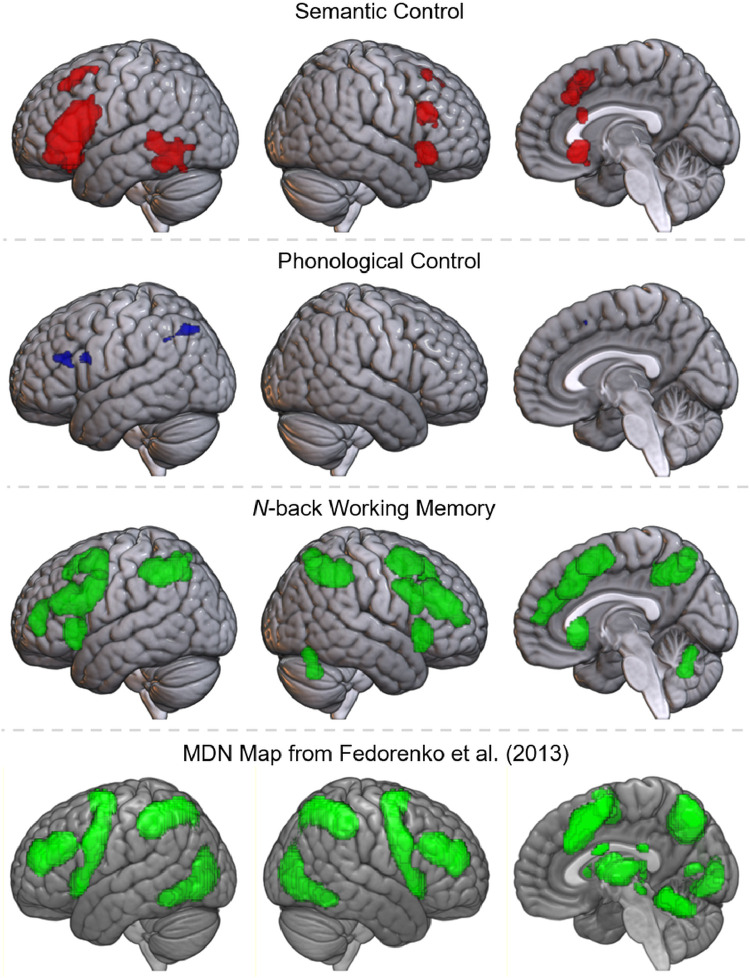
Top row: activation map of semantic control (red). Second row: map of phonological control regions (blue), represented by hard > easy phonology contrast. Third row: activation map for the *n*-back working memory domain. Bottom row: map of the multiple demand network from Fedorenko et al. (2013).

The *n*-back working memory ALE analysis yielded a distributed bilateral network for domain-general control (Fig. 4 & Table 9), highly similar to the mask from Fedorenko et al. (2013), although lacking temporal and occipital involvement. There are three clusters in bilateral dorsolateral prefrontal cortex, extending into precentral and inferior frontal gyri, and two in left and right insula, with some involvement of pars orbitalis. Further clusters are located in the inferior parietal lobule, extending dorsally and medially into the superior parietal lobule, and the dmPFC, right cerebellum and left thalamus. Both these results and the *a priori* MDN map overlap substantially with the regions implicated in both phonological and semantic control, but not the regions implicated in semantic control alone. Thus, the language network may include some regions implicated specifically in semantic control, or the manipulation of meaningful representations (i.e., ventral IFG and posterior MTG), and some regions responsible for domain-general control (i.e., dmPFC, dorsal IFG/dlPFC, posterior ITG, precentral gyrus and inferior parietal cortex).

**Table 9:** N-back working memory activation likelihood

Cluster	Region of Activation	Maximum ALE Value	Z Score	Peak MNI Coordinate		
				x	y	z
1	Left middle frontal gyrus/precentral gyrus	0.080	9.437	-44	8	32
		0.059	7.583	-44	30	24
		0.051	6.778	-36	52	10
		0.049	6.632	-30	2	56
		0.027	4.113	-56	20	24
		0.023	3.599	-50	16	6
2	Right middle frontal gyrus/precentral gyrus	0.078	9.253	44	42	24
		0.071	8.623	46	34	26
		0.044	6.068	52	12	30
3	Right inferior parietal lobule	0.107	11.62	44	-46	44
		0.064	8.003	34	-58	48
		0.044	6.111	16	-66	58
4	Bilateral dorsomedial prefrontal cortex	0.089	10.227	0	16	48
5	Left inferior parietal lobule	0.077	9.199	-36	-48	44
		0.071	8.654	-42	-44	46
		0.026	3.990	-12	-68	56
		0.024	3.738	-4	-60	54
6	Right middle frontal gyrus (dorsal)	0.071	8.644	28	8	58
7	Left insular/inferior frontal gyrus	0.092	10.423	-32	22	0
8	Left thalamus	0.042	5.877	-14	0	10
		0.040	5.633	-12	-6	10
9	Right insula/inferior frontal gyrus	0.090	10.290	34	24	-2
10	Right posterior cerebellum	0.037	5.314	32	-64	-32
		0.032	4.800	34	-68	-20

## 4 Discussion

**Fig. 5:**
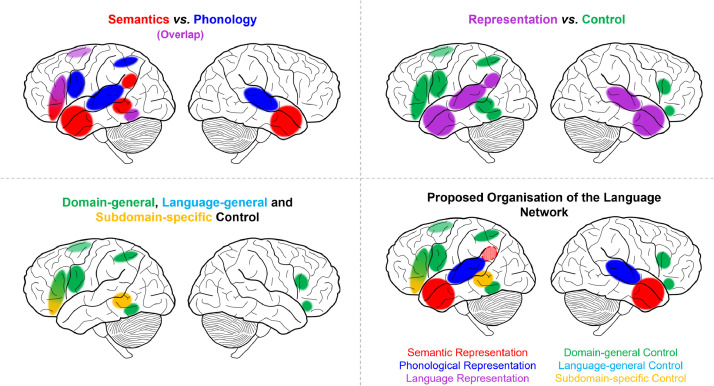
Schematic diagrams of the core organizational principles of the language network, based on the meta-analytic results. Top left: regions implicated in the semantic (red) and phonological (blue) subdomains with overlap shown in purple. Top right: regions implicated for semantic/phonological representation (purple) and control (green). Bottom left: domain-specificity of regions implicated in semantic and phonological control. Domain-general regions are shown in green and subdomain-specific regions in yellow (all of which reflect semantic control regions). Language-general regions would be displayed in light blue, yet no regions were found to be shared between semantics and phonology without a more domain-general role. Bottom right: a proposed multidimensional organization of the language network. The angular gyrus is shaded as its role in semantic representation is highly debated (Humphreys et al., 2015, Cabeza et al., 2012, Seghier, 2013); see Discussion for more detail.

A multidimensional approach is necessary to describe the underlying functional organization of the language network. Formal analyses confirmed prior hypotheses that distinguishing semantic and phonological subdomains provides one key organizational principle. Yet this domain-based separation alone is not sufficient. Distinguishing the processes of representation and control provides additional, distinct indications of the functional roles of regions throughout the language network. Consistent with prior assessments, the semantic network comprises the bilateral ATL for representation, alongside control regions in the left IFG, bilateral dmPFC and left posterior lateral temporal lobe (Jefferies, 2013, Patterson and Lambon Ralph, 2016, Binder et al., 2009, Jackson, 2021). The AG was also implicated in semantic representation, although there is ongoing debate as to whether this region truly contributes to semantic cognition overall, underpins a particular aspect of semantic cognition, or is identified solely based on confounding factors, such as difficulty-related deactivations (Humphreys et al., 2015, Cabeza et al., 2012, Seghier, 2013, Humphreys et al., 2020). It should also be noted that the present analysis did not attempt to separate lexical and conceptual semantic processing, held to be separable processes by many accounts (Levelt, 1992, Levelt, 2001, Levin and Pinker, 1991). Additionally, there may be further linguistic processes not delineated here, such as syntax. There is ongoing debate about whether syntactic processing engages distinct regions from semantics and phonology and how best to separate these processes (Fedorenko et al., 2020, Bornkessel-Schlesewsky et al., 2015, Mollica et al., 2020), which requires further research. We do, however, demonstrate here that the semantic regions are not simply syntactic*.* Phonological representation is supported by bilateral STG with control dependent on the left supramarginal and superior angular gyri, dorsal IFG/dlPFC and posterior lateral temporal cortex, as well as the dmPFC. The left precentral gyrus is identified in the phonological control contrast at lower thresholds (Supplementary Figure 2), and thus may also be involved in phonological control, perhaps specifically of complex motor sequences including those needed for articulation (Turkeltaub et al., 2002, Halai et al., 2017, Baldo et al., 2011), or in working memory more broadly (Lycke et al., 2008, Cabeza and Nyberg, 2000). The findings for the phonology domain are highly consistent with prior assessments of the phonological network (Poldrack et al., 1999, Buchsbaum et al., 2001, Hickok and Poeppel, 2007, Buchsbaum et al., 2011, Graves et al., 2008, Humphreys et al., 2015), yet provide additional evidence regarding the nature of the processing in these regions. Whilst the semantic and phonological representation regions are largely dissociable, there are both shared (left dorsal IFG, right IFG, left posterior inferior temporal cortex, precentral gyrus and bilateral dmPFC) and distinct (left ventral IFG and posterior MTG for semantic control only) control regions. The shared regions are all implicated in domain-general executive functions, forming part of the multiple demand network (Duncan, 2010, Camilleri et al., 2018, Assem et al., 2020). Semantic control has its own distinctive neural correlates, yet effortful tasks in both semantic and phonological domains recruit additional domain-general frontal and posterior inferior temporal control regions. This highlights the importance of a third factor; the domain-specificity of the regions recruited for language tasks. The language network is multi-faceted and only by considering the interactions between subdomain, process and domain-specificity, can we understand the function of an individual region. The contribution of multiple dimensions to the function of each region is demonstrated in a proposed schematic for the organization of the language network, presented in Fig. 5, based on the present meta-analyses in the context of the broader literature. The remainder of the Discussion will first describe important methodological considerations for future work before delineating the key implications for existing theories of organization within the language network.

Activation likelihood estimation is a powerful meta-analytic tool. Visual analysis of the distribution of peaks is clearly insufficient to determine the regions involved in a task or compare two distributed networks. However, it is the difference between the two ALE analyses that may be more surprising. Despite claims of distinct distributions, the Vigneau et al., 2006, Vigneau et al., 2011 datasets are not adequate to formally identify a strong separation between semantic and phonological processing. These datasets included 44 contrasts and 70 contrasts for semantics and phonology respectively, far higher than the recommended 17–20 contrasts minimum for a neuroimaging meta-analysis (Müller et al., 2018, Eickhoff et al., 2016). Although both within and between domain results varied by dataset, researchers should be particularly cautious when using datasets of this size to perform contrasts between meta-analysis results, which may require substantially more data. Separating phonology and semantics benefited from the increase in power brought about by the addition of a decade and a half of additional functional neuroimaging research. Of course, the simple amount of data may not be the critical issue, but the increase in data quality across time. More recent papers generally reported larger samples of participants and made use of a wide range of improved fMRI techniques.

Many models of the language network focus on the separation of subdomains as the single organizing principle (Catani et al., 2005, Friederici, 2009, Price, 2000, Price, 2012). For example, the dual-stream model of language, which proposes a largely bilateral ventral stream for lexico-semantic access and a left-lateralized dorsal stream for mapping sound to meaning, broadly divides the network into phonological and semantic streams (Hickok and Poeppel, 2007, Hickok and Poeppel, 2004, Saur et al., 2008, Ueno and Lambon Ralph, 2013). The identification of differences in ATL and left IFG aligns well with this dorsal-ventral division, as does the pattern of activation across hemispheres. Of course, such models do not attempt to capture the relationship between representation and control, or the overlap between language and domain-general regions. Yet it is the large amount of overlap between semantic and phonological processing that may be surprising from a dual-stream perspective, or on the basis of any unidimensional, process-based account. Divisions between other subdomains (e.g., syntactic processing, processing of sequences) may extend the current findings (Bornkessel-Schlesewsky et al., 2015, Matchin and Hickok, 2020), yet the evidence for a strong behavioural, cognitive or neural separation between these processes is not as clear (Friederici, 2002). Instead, a full consideration of function may require consideration of multiple dimensions focused on different kinds of information, including process and involvement of these regions outside language tasks. This may be understood in terms of the primary systems hypothesis; language processes are performed by combining the necessary domain-specific representation regions with the appropriate control regions (Patterson and Lambon Ralph, 1999, Woollams, 2014).

Language and executive function are often considered behaviorally and neurally independent, due to the ‘special’ nature of language (Fedorenko et al., 2012, Fedorenko and Blank, 2020). Yet areas typically associated with language may have more domain-general roles and multiple lines of evidence suggest interaction between these processes. For instance, after cerebrovascular accident, executive function is an important predictor of aphasia therapy outcome (Ralph et al., 2010) and there is increased reliance on the dmPFC for speech (Geranmayeh et al., 2017, Sliwinska et al., 2017). A large body of neuropsychological (Jefferies and Lambon Ralph, 2006, Rogers et al., 2015), neuroimaging (Noonan et al., 2013, Jackson, 2021) and neuro-stimulation (Whitney et al., 2011, Whitney et al., 2011) work now supports the importance of control processes within the semantic subdomain (see Lambon Ralph et al., 2017, Jefferies, 2013 for a more detailed review). Our results indicate the need to examine the same distinction within the phonology subdomain. Only the superior temporal gyri was specifically implicated in phonological processing. All other regions implicated in phonology were also involved in domain-general control, including dorsal IFG/dlPFC, inferior parietal lobe, precentral gyrus, dmPFC and posterior ITG. Whilst language researchers may not expect temporal regions to be implicated in control (typically thought to rely on frontal regions), the present finding that pMTG is involved in semantic control aligns with an increasing wealth of patient, neuroimaging and TMS data (Noonan et al., 2013, Jefferies and Lambon Ralph, 2006, Noonan et al., 2009, Whitney et al., 2011, Whitney et al., 2011); meanwhile, the posterior ITG has received increasing recognition in the executive function literature (and is not simply related to the presentation of visual stimuli) (Assem et al., 2020, Fedorenko et al., 2013, Woolgar and Zopf, 2017). Thus, there may be two distinct regions of posterior temporal cortex, responsible for semantic-specific and domain-general control processes or a graded transition within a control region with different specialties. The role of the pITG is hard to align with models of phonological processing that associate these regions with phonology-specific processes, such as orthographic-phonologic mapping or speech segmentation (Poldrack et al., 1999, Burton, 2001). It should be noted that posterior ITG may still reflect language-specific control, as it was implicated in both phonological and semantic control, was not implicated in nonverbal working memory, and has been found in MDN assessments which did not specifically exclude verbal stimuli (Assem et al., 2020, Fedorenko et al., 2013). However, this region has been implicated in non-verbal executive function, such as task switching in both patients and neuroimaging (Buchsbaum et al., 2005, Kim et al., 2012, Schumacher et al., 2019); therefore, it may be more likely that the pITG is instead responsible for a domain-general function distinct from working memory, perhaps related to task-shifting or attention. What particular control processes might be critical for phonological processing? The term ‘phonological control’ has previously been associated with phonological working memory in the form of the articulatory loop (Baddeley et al., 1984, Baddeley and Hitch, 1994, Clark and Wagner, 2003). However, working memory alone cannot explain the full distribution of the current results. For instance, posterior ITG was not implicated in working memory. Moreover, regions across the MDN are found to have a role across tasks requiring different forms of executive control (Camilleri et al., 2018, Assem et al., 2020). Thus, phonological control may require a range of executive functions as with semantic control, perhaps reflecting similar elements, such as selection between possible words, inhibition of alternatives and attention shifting. It should be noted that, unlike semantic control, phonological control was assessed with a comparison across studies due to the lack of studies directly manipulating control in phonology. The current highlights the need to consider and directly manipulate control requirements in future studies of phonology. This distinction between control and representation may be critical to understand disorders associated with phonology, such as developmental dyslexia, which has been demonstrated to reflect an access problem and not simply a representational deficit (Ramus, 2014, Boets et al., 2013). Indeed, the effects of damage to these regions post-stroke have been dissociated, with phonological representation regions having distinct behavioral effects compared to damage to the regions here designated as control (Halai et al., 2017, Schumacher et al., 2019, Catani et al., 2013, Lacey et al., 2017). The current findings highlight the need to consider and directly manipulate control requirements in future studies of healthy and impaired phonological processing.

Unlike phonological control, not all of the areas implicated in semantic control are responsible for domain-general control. Ventral inferior frontal and posterior middle temporal regions were not identified as part of the MDN, in keeping with prior research (Duncan, 2010, Assem et al., 2020). Instead, these regions appear specialized for the control of meaningful stimuli. Indeed, a specific impairment of semantic control results from damage to these particular regions in semantic aphasia (Jefferies and Lambon Ralph, 2006, Berthier, 2001). The control of such meaningful multimodal stimuli may heavily rely on particular control processes; for instance, the left IFG – the ventral part of which is found here to be a domain-specific control region – may perform competition selection and suppression (Jefferies, 2013, Badre et al., 2005, Davey et al., 2016, Corbett et al., 2010, Corbett et al., 2009, Moss et al., 2005, Zhang et al., 2004), or the unification of language representations (Hagoort, 2013, Hagoort, 2005). This may align with our hypothesis that the most ventral part of left IFG is a domain-specific semantic control region, while dorsal IFG/dlPFC constitute multiple demand areas that are recruited for controlled language tasks. Why, if language as a whole is not ‘special’, might semantic cognition recruit unique control regions? These regions are critical for the context-dependent access and manipulation of all meaningful items, including pictures, objects, faces and environmental sounds, as well as language (Lambon Ralph et al., 2017). The control of such meaningful multimodal stimuli may heavily rely on particular control processes, such as competition selection and suppression (Jefferies, 2013, Badre et al., 2005, Davey et al., 2016, Corbett et al., 2010, Corbett et al., 2009). Alternatively, the process may be equivalent across control regions, yet the connections of these particular areas may be conducive to the application of control to meaningful stimuli, for example, due to the nature of their connections with the anterior temporal lobe hub. Indeed, it is not clear whether these distinctions ought to be viewed as distinct regions for semantic and domain-general control or graded changes within a larger complex (Jackson, 2021, Assem et al., 2020). Semantic control regions lie adjacent to regions implicated in more domain-general control, including ventral versus dorsal IFG and posterior middle versus inferior temporal gyri. Further research should consider to what extent these reflect graded changes in function versus a sharp shift between distinct functional regions.

## Declaration of Competing Interest

The authors declare no competing interest.

## Appendix Supplementary materials

Image, application 1
